# ‘Practice what you preach’: Nurses’ perspectives on the Code of Ethics and Service Pledge in five South African hospitals

**DOI:** 10.3402/gha.v8.26341

**Published:** 2015-05-11

**Authors:** Janine White, Maureen Phakoe, Laetitia C. Rispel

**Affiliations:** 1 School of Public Health, Faculty of Health Sciences, University of the Witwatersrand, Johannesburg, South Africa; 2 Centre for Health Policy & Medical Research Council Health Policy Research Group, School of Public Health, Faculty of Health Sciences, University of the Witwatersrand, Johannesburg, South Africa

**Keywords:** code of ethics, nurses pledge, nurses, ethical dilemmas, South Africa

## Abstract

**Background:**

A recent focus of the global discourse on the health workforce has been on its quality, including the existence of codes of ethics. In South Africa, the importance of ethics and value systems in nursing was emphasised in the 2011 National Nursing Summit.

**Objective:**

The study explored hospital nurses’ perceptions of the International Code of Ethics for Nurses; their perceptions of the South African Nurses’ Pledge of Service; and their views on contemporary ethical practice.

**Methods:**

Following university ethics approval, the study was done at a convenience sample of five hospitals in two South African provinces. In each hospital, all day duty nurses in paediatric, maternity, adult medical, and adult surgical units were requested to complete a self-administered questionnaire. The questionnaire focused on their perceptions of the Code of Ethics and the Pledge, using a seven-point Likert scale. STATA^®^ 13 and NVIVO 10 were used to analyse survey data and open-ended responses, respectively.

**Results:**

The mean age of survey participants (*n*=69) was 39 years (SD=9.2), and the majority were female (96%). The majority agreed with a statement that they will promote the human rights of individuals (98%) and that they have a duty to meet the health and social needs of the public (96%). More nuanced responses were obtained for some questions, with 60% agreeing with a statement that too much emphasis is placed on patients’ rights as opposed to nurses’ rights and 32% agreeing with a statement that they would take part in strike action to improve nurses’ salaries and working conditions. The dilemmas of nurses to uphold the Code of Ethics and the Pledge in face of workplace constraints or poor working conditions were revealed in nurses’ responses to open-ended questions.

**Conclusion:**

Continuing education in ethics and addressing health system deficiencies will enhance nurses’ professional development and their ethical decision-making and practice.

A recent focus of the global discourse on the health workforce has been on its quality, including evidence of regulatory bodies in countries and the existence of codes of ethics (1). Ethical standards are common in the healthcare professions and are operationalised through codes of ethics and/or service pledges (2, 3). A code of ethics governs professional conduct and is a symbolic written expression, whereas a pledge is the verbal expression of ethical considerations in professional conduct (4). The code of ethics and practice of pledges have their origins in the deontological view that it is a nurse's responsibility to act in the best interest of individuals in their care. This view suggests that professional values influence nurses’ behaviour in practice, and assumes that there is a direct link between nurses’ awareness and understanding of the code and pledge and their ethical behaviour and practice (4, 5).

The International Code of Ethics for Nurses was developed in 1953 by the International Council for Nurses (ICN) (6). The Code has undergone four revisions, with the most recent revision in 2012. The ICN Code is organised into four fundamental guiding elements: nurses and people, nurses and practice, nurses and the profession, and nurses and co-workers (6). The first element, nurses and people, focuses on nurses’ interactions with people in the healthcare setting. It upholds the advancement of the human rights of individuals, family, and community; the dissemination of information for decision-making; and the protection of confidential information (6). The second element, nurses and practice, links professional nursing practice with personal responsibility and accountability, and highlights the importance of continuing education (6). The third element, nurses and the profession, emphasises the nurse's role in the implementation of acceptable standards of clinical practice, while the fourth element, nurses and co-workers, is concerned with the relationship between the individual nurse and other co-workers, the protection of individuals, families, and communities when a health threat is identified, and the promotion of ethical conduct among co-workers (6).

The International (Nightingale) Nurses’ Pledge is a promise undertaken by nurses to uphold the core values and principles of nursing, and is aligned to the ICN Code of Ethics. It refers to promoting the rights of patients and the promotion of health, protection of information, refraining from endangering the life and health of patients, maintaining professional competence and continuing education, and sustaining good working relationships with colleagues (7). The Pledge is commonly recited at nursing graduation ceremonies around the world, including in South Africa.

In South Africa, the South African Nursing Council (SANC) Pledge of Service (also referred to as the Nurses’ Pledge or the Pledge) is an adapted version of the Nightingale Pledge (8). The SANC, established in terms of the Nursing Act (9), is the regulatory body for all categories of nurses (including midwives) and is responsible for setting standards for professional and ethical nursing practice, and nursing education and training throughout South Africa (10). Hence, the education of all professional nurses with 4 years of training includes a mandatory ethics component, prior to registration with the SANC.

The importance of ethics and value systems in nursing was emphasised at the 2011 National Nursing Summit, that brought together close to 2000 nurses in South Africa (11). The final report of the Ministerial Task Team on Nursing Education, Training and Practice contains key recommendations on ‘restoring ethics and respect in nursing’ and the ‘mainstreaming of ethics in all nursing training programmes’ (11).

Notwithstanding the importance of ethics and value systems in nursing, the Code of Ethics and Nurses’ Pledge are not without controversy, particularly in the context of industrial action by nurses to achieve enhanced status for the profession, improved career paths, increased salaries, and better quality of care for patients (12–16). Nonetheless, there are several studies that have focused on Codes of Ethics for nurses in different country contexts (14, 17–23), ethical dilemmas faced by nurses in the workplace (5, 24–28), and the ethical issues involved in industrial action by nurses (12, 13, 15, 16, 29, 30). Those studies that have focused on Codes of Ethics have found that there is widespread support among nurses as these Codes serve as a guide to ethical decision-making and behaviour (14, 17–22). However, some scholars have pointed out that these Codes do not assist with ethical dilemmas in the workplace or with solving the resource constraints and poor working conditions faced by nurses (14, 18, 24, 31). This is exacerbated by lack of awareness of Codes of Ethics and sub-optimal knowledge among nurses of what constitutes good ethical practice (19, 21, 31, 32).

In South Africa, a number of studies have focused on the tensions between nurses’ ethical values or conduct and industrial action (12, 29, 30), or ethical values and termination of pregnancy (28). In light of the renewed focus of the South African government on the nursing profession and the call for the implementation of ‘a comprehensive programme to restore ethics and respect in nursing’ (11), the aim of this study was to explore nurses’ perspectives on the ICN Code of Ethics and the SANC Nurses’ Pledge in a convenience sample of hospitals in Gauteng and Free State Provinces of South Africa.

## Methods

The study was nested in a larger project that examined the nature and dynamics of nursing management and quality of care in hospitals. The larger study focused on nine randomly selected hospitals in Gauteng (urban) and Free State (mixed urban–rural) Provinces in South Africa. The choice of the two provinces, Gauteng and the Free State, was purposive, and influenced by geographical proximity to the researchers, prior health authority approval, and budgetary constraints. Gauteng is the most urbanised and densely populated province with a population of 12.2 million, while the Free State Province is a mixed urban–rural, largely agricultural province, with a population of 2.7 million (33).

Because the larger study was already underway when the ethics protocol was finalised, the ethics component of the study was done in the two remaining private hospitals in Gauteng Province, and the three public hospitals in Free State Province. Hence the five hospitals, although randomly selected as part of the larger study, could be considered as a convenience sample for the ethics study.

The Human Research Ethics Committee (Medical) of the University of the Witwatersrand in Johannesburg provided ethics approval for the study. The relevant public and private health care authorities also gave the necessary study approvals. All participants received a study information sheet and provided written, informed consent.

At each hospital, one medical, surgical, paediatric, and maternity (labour and post-natal) unit was selected (total four units per hospital); hence, 20 units were part of the study. Emergency and critical care units were excluded because the focus of the larger study was on nursing unit managers. In the ethics study, the population of interest was all categories of nurses registered with the SANC. On the survey day, all day duty nurses working in the selected units were invited to participate in the study. Night duty nurses were excluded because of logistical difficulties in conducting fieldwork at night at the selected hospitals.

Following informed consent, each nurse completed a self-administered questionnaire that contained both closed and open-ended questions. The questionnaire consisted of four sections. The first three sections contained closed-ended questions that elicited information on: participant characteristics; perspectives on the ICN Code of Ethics; and perspectives on the South African Nurses’ Pledge of Service. The Code of Ethics section in the questionnaire consisted of 14 questions, which related to each of the four elements of the ICN Code (6). The section on the Nurses’ Pledge consisted of nine questions, which related to the elements of the Pledge (7). The questions were designed on a seven-point Likert scale ranging from 1 (strongly disagree) to 7 (strongly agree). The questions on the Nurses Code of Ethics and Nurses’ Pledge were phrased in a manner that attempted to minimise an unreflective response by participants. The fourth section consisted of three open-ended questions that focused on the opinions of participants on current ethical practice of nurses, strategies for enhancing ethical practice, and any other comments on the Code of Ethics or Nurses’ Pledge. The questionnaire was piloted prior to implementation, and no changes were deemed necessary.

We analysed the quantitative data using STATA^®^ 13. Frequency tabulations were done to describe the socio-demographic characteristics of the respondents, and the responses to questions on the Nurses’ Code of Ethics and Nurses’ Pledge. A thematic content analysis of transcripts was conducted (34) using NVIVO version 10 software for the qualitative data analysis. Two members of the research team (LCR and JW) coded the open-ended questions independently and then established inter-coder agreement. Once the codes were agreed to, the transcripts were loaded into the software, and the coding of each transcript was done to identify recurring themes. To ensure the trustworthiness of the data, continuous peer debriefing and checking of researchers’ interpretations against the raw data was done.

## Results

The study recruited 69 nurses of all categories in the five hospitals. There were no refusals, representing a 100% response rate.

### Participant characteristics

The age of participants ranged from 23 to 61 years, with a mean age of 39 years (SD 9.2). The majority of participants were female (96%) and employed in public hospitals (61%) (Table 1).

**Table 1:** Demographic and employment characteristics of study participants

Characteristic	Total^a^
Number of participants	69
Mean age (standard deviation)	39 (9.2)
<29 years (%)	16 (23)
30–39 (%)	20 (29)
40+ years (%)	33 (48)
Sex	
Female (%)	66 (96)
Male (%)	3 (4)
Marital status	
Married (%)	32 (46)
Living together (%)	4 (6)
Single (%)	28 (41)
Divorced/separated (%)	1 (1)
Widowed (%)	4 (6)
Category of nurse	
Professional nurse (%)	23 (35)
Enrolled nurse (%)	13 (19)
Auxiliary nurse (%)	17 (25)
Category not specified	15 (22)
Sector of employment	
Provincial hospital (%)	42 (61)
Private hospital (%)	27 (39)
Unit of work	
Paediatric (%)	9 (13)
Medical (%)	17 (25)
Surgical (%)	17 (25)
Maternity (%)	19 (27)
Other (%)	5 (7)

a Minor discrepancies due to missing values.

### Nurses’ opinions on the ICN Code of Ethics

Table 2 shows the participants’ opinions on the 14 questions that explored their opinions on the ICN Code of Ethics, ranked in order of the level of agreement.

**Table 2:** Nurses’ opinions on the ICN Code of Ethics

Statement	*n* ^a^	Agree %	Neutral %	Disagree %
I play an active role to maintain good relationships with my co-workers	69	100	0	0
I promote the human rights of individuals under my care	66	98	2	0
I always maintain the standards of personal conduct required by my profession	69	96	0	4
I have a duty to meet the health and social needs of the public	68	96	1	3
I get upset when I do not have equipment to provide good patient care	68	95	0	5
I think too much emphasis is placed on patients’ rights at expense of nurses’ rights	65	60	10	30
I will take part in strike action to improve nurses’ salaries	68	32	6	62
I believe that a trade union is better than a professional organisation to improve nurses’ socio-economic conditions	68	26	19	55
I would provide information on a patient's HIV positive status to his/her family	69	22	2	76
It is not my responsibility to change the image of nursing	68	19	2	79
Patients should not receive a lot of information about their care as it confuses them	65	17	2	81
It is not my role to implement acceptable standards of clinical nursing practice	69	10	2	88
Providing care to homosexuals is against my ethical values	67	6	8	86
It is not my own responsibility to maintain professional competence	66	6	0	94

a Discrepancies due to missing values.

As can be seen from the table, 100% of respondents agreed with the statement ‘I play an active role to maintain good relationships with co-workers’. High levels of agreement were found for promotion of the human rights of the people in their care (95%); the nurses’ duty to meet the health and social needs of the public (96%); maintaining the standards of personal conduct required by the nursing profession (96%); and ‘getting upset’ when lacking necessary equipment to provide good patient care (95%).

Similarly, high levels of disagreement were found in negative statements such as ‘nurses do not have a responsibility to maintain professional competence’ (94%), ‘it is not my role to implement acceptable standards of clinics nursing practice’ (88%), ‘providing care to homosexuals is against my ethical values’ (86%) and ‘patients should not receive a lot of information about their care as it confuses them’ (81%). More nuanced responses were obtained for the three questions that elicited responses on nurses versus patients’ rights, strike action, and a trade union versus a professional association (Table 2).

### Nurses’ opinions on the Pledge of Service

Table 3 shows the participants opinions on the nine questions that explored their opinions on the Nurses’ Pledge, ranked in order of the level of agreement.

**Table 3:** Nurses’ opinions on the Pledge of Service

Statement	*n* ^a^	Agree %	Neutral %	Disagree %
I care for sick patients with all the skill and understanding I possess	66	100	0	0
I recited the Nurses’ Pledge with pride	66	98	0	2
I make efforts to keep my professional knowledge and skills at the highest level	68	98	0	2
I hold in confidence all personal information given over to me	65	93	0	7
I am passionate about alleviating suffering	67	77	3	20
I cannot uphold the integrity of the professional nurse	66	17	0	83
Race influences the way I take care of patients	66	9	3	88
I cannot respect the religious beliefs of patients under my care	68	3	0	97
People who pay for their care should get better services than those who do not pay	68	3	3	94

a Discrepancies due to missing values.

As can be seen from Table 3, 100% of respondents agreed with the statement ‘I care for sick patients with all the skill and understanding that I possess’. High levels of agreement were found for statements on reciting the Nurses’ Pledge with pride (98%); and making effort to keep the highest level of professional knowledge and skills (98%).

Similarly, high levels of disagreement were found in the negative statement ‘I cannot respect the religious beliefs of patients under my care’ (97%) and that ‘People who pay for their care should get better services than those who do not pay’ (94%).

### Opinions on contemporary ethical practice of nurses

Although overlapping, three broad themes emerged from nurses’ responses to the open-ended questions. These were: insufficient awareness or knowledge on what constitutes good ethical practice; the need for a strong ethics focus in nursing education and training; and the dilemma of nurses to uphold the code of ethics and service pledge in face of workplace constraints or poor working conditions.

The majority of participants were of the opinion that there is insufficient awareness or knowledge among students or younger nurses on what constitutes good ethical practice.

Nurses of today have got no clue of the word ethics. One could see with the way they lack professionalism, etiquette and confidentiality. [Respondent 13, Gauteng private hospital 2]

The Nurses’ Pledge is only for registered or professional nurses, other categories of nursing do not know about it, but they are also practising as nurses. They should also be taught about ethics and pledge. [Respondent 57, Free State public hospital 2]

The study participants were also of the opinion that nursing education and training programmes should place a greater emphasis on ethical behaviour and practice.

The ethical practice must be improved. All [nurses] must know about the rules and regulations and the Code of Ethics. [Respondent 21, Gauteng private hospital 2]

I think they must start in the colleges, the learning centres – they must start to tell these people what is ethics, because I don't think they know – they write exams, but I don't think they know how to practise this in the operational field. [Respondent 37, Free State public hospital 1]

Training and education on ethical practice in nursing should be done regularly to all nursing personnel, maybe after every six months. [Respondent 51, Free State public hospital 2]

Ethical dilemmas mentioned by study participants included: providing care in the face of disrespect from patients, the problems created by individuals who do nursing for the wrong reasons; and being blamed by managers for errors or mistakes, despite staff shortages or health system deficiencies, especially in public hospitals.

Some respondents were of the opinion that patients have a ‘bad attitude towards nurses’, and their disrespectful behaviour, makes it more difficult for nurses to practise ethically. Several study participants lamented about the perceived ‘poor’ calibre of new entrants to the nursing profession, as can be seen from the following comments:

New nurses’ attitudes are completely different than 20 years ago. There is need for a complete turnaround and thorough sifting of individuals before they enter the [nursing] profession. [Respondent 9, Gauteng private hospital 1]

I think the problem lies with the nurses that start to nurse for the wrong reasons – nursing has become a money-spinning thing, but the deep passion and that deep born thing for caring is missing. [Respondent 37, Free State public hospital 1]

Some study respondents, particularly in public hospitals, mentioned health system deficiencies as hindering ethical practice by nurses.

The ethical practice of nurses is not up to standard due to shortage of staff. Hire more staff to boost nurses’ morale and reduce stress periods. [Respondent 38, Free State public hospital 2]

People lose [their] morale in the nursing profession. There must be enough staff, equipment, and nurses must get enough support from the government–financially and psychologically. [Respondent 40, Free State public hospital 2]

### Strategies to enhance ethical practice in nursing

The overwhelming number of responses related to a ‘practice what you preach’ approach to the Code of Ethics and the Pledge, illustrated by the comment below:

Nursing students should practise professionalism at an early level in the college or university. All nursing educators and registered nurses must be role models for nursing students and other subordinates, such as enrolled nurses. [Respondent 64, Free State public hospital 3]

Recommendations included the need for continuous professional education on ethical behaviour and practice; using health service events (e.g. hospital open days) to remind nurses of good ethical practice; and addressing nurses’ salaries and working conditions, resource constraints and health system deficiencies such as the lack of functioning equipment.

## Discussion

This study found that there were high levels of awareness among nurses in the selected hospitals on the Code of Ethics and the Pledge, illustrated by their responses to a series of proxy statements on the Code of Ethics and Nurses’ Pledge. The questions on the Code of Ethics (Table 2) elicited high levels of agreement on the statements regarding the maintenance of good relationships with co-workers, the promotion of the human rights of patients, the nurses’ duty to meet the health and social needs of the public, maintaining the standards of personal conduct required by the nursing profession, and ‘getting upset’ when lacking necessary equipment to provide good patient care. Similarly, high levels of disagreement were found in negative statements where such disagreement was appropriate. The statements on the Nurses’ Pledge of Service (Table 3), also found high levels of agreement on caring for sick patients with the necessary skill and understanding, reciting the Nurses’ Pledge with pride; and making effort to keep the highest level of professional knowledge and skills. Although not directly comparable, other studies have also found high levels of awareness of and support for Codes of Ethics to guide ethical decision-making and behaviour (14, 17–22).

More nuanced responses were obtained for the three questions that elicited responses on nurses versus patients’ rights, strike action, and a trade union versus a professional association. The majority of respondents (60%) agreed with the statement ‘I think too much emphasis is placed on patients’ rights at the expense of nurses’ rights’. This was borne out by the qualitative comments made in the open-ended questions, with some nurses indicating that perceived disrespect from patients influenced their ability to provide optimal quality of care. The finding could reflect the emphasis on the government's core national standards that aim to improve patients’ experiences of public sector care at the time of the study (35), and misunderstanding on the part of nurses regarding the overall goal of the national core standards.

Although a minority of nurses agreed with the statements that they will embark on strike action to improve nurses’ salaries (32%) and that a trade union is better than professional association to improve the socio-economic conditions of nurses (26%), the results are not surprising. Another South African study also found that 32.5% of study participants supported strike action by nurses as a constitutional and legal right (29). Studies in other countries have also found that although strike action is not an easy decision, nurses will embark on industrial action to achieve improvements in the health care system and in their own working conditions (13, 15).

The three themes that emerged from the analysis of the open-ended questions: insufficient awareness or knowledge on what constitutes good ethical practice; the need for a strong ethics focus in nursing education and training; and the dilemma of nurses to uphold the Code of Ethics and the Pledge in the face of workplace constraints or poor working conditions contradicted the participants’ responses to the closed-ended questions. There are several reasons for this apparent contradiction. Firstly, the responses might be a reflection of social desirability bias (36), with participants giving responses that they thought the researchers wanted to hear, or that were appropriate at the time of the study. Secondly, the contradictory responses might reflect the disjuncture between their levels of awareness and the difficulties of translating that awareness or knowledge into practice. Thirdly, it might be that ethics is dealt with in pre-service nursing training, but that there is little focus and discussion on dealing with ethical issues in the workplace.

This was one of the first studies in South Africa to explore nurses’ views on the ICN Code of Ethics and the Nurses’ Pledge, following the 2011 National Nursing Summit in the country. The paper makes an important contribution to the discourse on ethical behaviour and practice of nurses and midwives. However, the findings are not generalisable as the study was small and limited to five South African hospitals that constituted a convenience sample. Although the statements that served as a proxy to determine nurses’ views on the Code of Ethics and the Pledge of Service were phrased carefully to avoid non-reflective responses, the contradictory responses indicate some social desirability bias (36). The cross-sectional design means that the study reflects the views of nurses at a point in time. Nonetheless, the study provides valuable insights into nurses’ perspectives on the International Code of Ethics and the Nurses’ Pledge of Service, and opens a scholarly discourse on ethics in South Africa. Further research on ethics is needed, which focuses on a more representative sample of nurses and which examines possible variations in the perceptions and ethical dilemmas faced by nurses between urban and rural areas, and between the public and private health sectors.

Notwithstanding the limitations of the study, the findings support the recommendations contained in the strategic plan on Nursing Education, Training and Practice (11). These recommendations include: raising awareness on ethics as a means to strengthen ethical nursing practice; ‘core, compulsory modules at all levels of nursing and midwifery training’ that emphasise professionalism, ethics and caring; and the introduction of a ‘Continuing Professional Development (CPD) system for all nurses and midwives, linked to licensing and professional progression, and which includes professionalism and ethics as a compulsory component’ (11, p. 7).

Other studies have also recommended raising awareness of professionalism and ethical behaviour as a means of strengthening ethical nursing practice (23, 37). Our study participants expressed concern with the quality and content of nursing ethics at undergraduate level. They recommended that greater emphasis should be placed on ethics at undergraduate level, but that ethics should be linked clearly to the competencies of nurses and their ability to provide good quality of care and navigate their way through ethical dilemmas. However, the SANC is the regulator of nursing and midwifery standards of practice, education and training in South Africa and emphasises the ethical and moral obligations of nurses in performing their duties (9). These obligations are expressed as Regulations to the Nursing Act (9). The participants’ perceptions of the inadequacy of ethics training in pre-service nursing education and training could be related to the policy-implementation gaps that are well described (38, 39). A study in Thailand found that appropriate didactic methods were necessary to create a learning environment that promotes ethical practice and appropriate behaviour of nursing students (37). The authors recommended additional training of new graduate nurses in decision-making and how to manage ethical dilemmas in nursing practice (37).

However, some scholars have pointed out that raising awareness is important, but that a holistic understanding of ethical practice includes the four components of ethical sensitivity, ethical judgement, ethical motivation and ethical action (5). Specific strategies recommended include CPD that focuses on the four component model, the establishment of nursing ethics groups, nurses’ participation in inter-disciplinary ethics rounds, the possible introduction of nursing ethics ward rounds, seeking guidance or support from senior or experienced nursing or medical colleagues, and a wider discourse on ethical nursing practice (5, p. 69).

Other nursing scholars have warned about the imposing position that pledges and oaths may bring (40) and have recommended a change leadership approach within nursing that combines visible, ethical leadership with participation of front-line nurses (40). Such leadership includes appropriate role modelling, which emphasises again the ‘practice what you preach’ approach, suggested by many study participants.

Importantly, the health system deficiencies alluded to by study participants would need to be addressed in order to facilitate ethical nursing practice. The appointment of the Chief Nursing Officer at the beginning of 2014 and a detailed strategic plan for Nursing Education, Training and Practice provide a good foundation in South Africa for strengthening ethics training and enhancing ethical nursing practice.

## Conclusion

In light of the numerical dominance of nurses in South Africa, and their role in the health and well-being of patients and communities, the importance of their technical and ethical competence is undisputed. The study participants displayed high levels of awareness of the ICN Code of Ethics and Nurses’ Pledge of Service. However, the responses revealed contradictions between knowledge and awareness, and contemporary nursing practice within a value-based, ethical framework. Continuing education in ethics and addressing health system deficiencies will enhance nurses’ professional development and their ethical decision-making and practice.
